# Food security and COVID-19 in Afghanistan: a two-sided battlefront

**DOI:** 10.1186/s41182-021-00370-8

**Published:** 2021-09-26

**Authors:** Attaullah Ahmadi, Georges Gandour, Hujjatullah Ghaffari, Heba Sati, Anthony Abou Mrad, Serena Semaan, Yasir Ahmed Mohammed Elhadi, Sayed Hamid Mousavi, Shamim Arif, Shekiba Madadi, Don Eliseo Lucero-Prisno

**Affiliations:** 1 Medical Research Center, Kateb University, Kabul, Afghanistan; 2 Department of Public Health, International School of Medicine, Bishkek, Kyrgyzstan; 3 Faculty of Medicine and Medical Sciences, University of Balamand, Koura, Lebanon; 4 Kabul University of Medical Sciences, Kabul, Afghanistan; 5 Faculty of Health Sciences, University of Balamand, Koura, Lebanon; 6 Department of Health Administration and Behavioral Sciences, High Institute of Public Health, Alexandria University, Alexandria, Egypt; 7 Department of Public Health, Medical Research Office, Sudanese Medical Research Association, Khartoum, Sudan; 8 Afghanistan National Charity Organization for Special Diseases (ANCOSD), Kabul, Afghanistan; 9 Harvard Medical School, Harvard University, Boston, MA USA; 10 Department of Global Health and Development, London School of Hygiene and Tropical Medicine, London, UK

**Keywords:** Food security, COVID-19, Pandemic, Challenges, Recommendations, Afghanistan

## Abstract

Food security is a major element for the sustainability, stability and development of a country. However, despite fundamental efforts in fighting poverty, Afghanistan continues to struggle with food insecurity with a large proportion of its population living below the poverty line. With extreme climates, protracted conflict, and now COVID-19, food insecurity has become rampant and is on the rise in the country. Efforts have been reinforced to mitigate this issue, but a spate of obstacles which seems not to come to an end, has contributed to the deterioration of the situation. With the collaboration and efforts of international organizations, there remains a glimmer of hope to potentially reduce the gravity of the food insecurity in the country. This paper aims to highlight the efforts of Afghanistan in alleviating food insecurity with a focus on the impact of COVID-19 on this issue. It also presents recommendations that may help ameliorate the country’s food security status during and after the pandemic.

## Introduction

Food insecurity is a looming crisis that has challenged almost a third of the world’s population [1]. It emerges when nutritional and food accessibility is not attainable [2]. Afghanistan has been struggling with acute and chronic food insecurity where 90% of its population live below the poverty line [3]. According to the United Nations Office for the Coordination of Humanitarian Affairs (UNOCHA), about 35% of its total population required urgent food and agricultural aid in 2020 [4]. This deteriorating situation of food security is not the result of a single cause but numerous challenges and factors including conflict, natural disasters and climate changes, economic downturn, and now COVID-19 [1, 4]. As COVID-19 has become the major public health concern of our time [5, 6], it is crucial to analyze its impact on socio-economic and food status besides health implications on the Afghan population. This paper aims to highlight the efforts of Afghanistan in alleviating food insecurity with a particular emphasis on the impact of COVID-19 on this issue. The paper also provides recommendations that may help improve the country’s food security during and after the pandemic.

## Commentary

As a country with one of the lowest scores on the Human Development Index (HDI) [4], Afghanistan happens to be adversely affected by a combination of factors impacting food security. One of the many contributors is the four decades of war which has dramatically affected organizations, irrigation, and agricultural infrastructures [4]. Continuing conflict and climate disasters work synergistically, thus burdening the progress in securing food. Natural disasters have contributed hugely to the nutritional status of the country. The arable land is considered to be climate-sensitive for agricultural production [1]. For instance, 70% of natural disasters in Afghanistan were floods, which left the inhabitants without adequate food [1]. Droughts influenced the agricultural infrastructure, thus decreasing nutritional supplies including crops and animal farm produce [7]. Several studies show that there is an inverse correlation between the cost of food and the loss of family income, drought, and food security in Afghanistan [4].

Considering Afghanistan’s low capacity to fight this pandemic, the health authorities, however, did not remain unresponsive to its food crisis**.** A major initiative done to tackle this issue was the humanitarian response provided by the Food Security and Agriculture Cluster (FSAC) aiming to secure food insurance to the most vulnerable [8]. Responses consisted mainly of agriculture and livestock kits which helped Afghan residents in guaranteeing their upcoming meals as well as empowering the rural workforce [8]. Alongside these reinforcements, multiple precautions were taken by the government to stop the spread of the virus including the closure of public areas. However, the necessary steps taken to prevent the infection led to more famine, poverty, and malnourishment since most of the population made a living through daily wages [9–11]. To help Afghans address the effects of the dry spells and COVID-19, the International Development Association (IDA) of the World Bank provided a grant of 97.50 million USD for the country to enhance food security [12]. This fund provided financial assistance to families to build resilience before and during dry seasons which will complement the task of the government in providing water, climate data management, warning systems, and fortifying preparation towards disasters within communities [12].

As of 2019, the World Food Program (WFP) stated that Afghanistan was undergoing one of the worst food crises [13]. With the arrival of COVID-19, Afghanistan’s situation was further exacerbated. The sudden onset of the pandemic was a major factor in aggravating acute food insecurity [14]. As one of the world’s hunger hotspots, there is a need for continuous flow and increased assistance to curb the increasing trend in the incidence of famine. To create data and provide some statistics, the Integrated Food Security Phase Classification (IPC) researched food insecurity amid COVID-19 in Afghanistan. In September 2020, 11.15 million people were reported to be in high level of acute food insecurity (IPC Phase 3 or more) with 3.6 million being in the Emergency phase (IPC Phase 4) [15]. These results represented a 3% increase from the numbers obtained the previous year during the same period showing the worsening situation.

COVID-19 did not just affect Afghans directly as it also caused the failure to provide sufficient employment together with income decline and higher food prices, thus compounding the circumstances around the pandemic [15]. About 15 million Afghans have also been reported to be “particularly vulnerable” to lockdowns as the number represents at least two million households across the country—most of whom are involved in activities such as shop keeping, street or market sales, or daily labor [16]. As a result, the World Bank predicted that poverty will increase in the country. Other factors are equally responsible alongside the pandemic. As Afghanistan is one of the most vulnerable and least equipped countries in the world to handle climate change [17], it has experienced several periods of drought, flood, avalanche, extreme weather and, as aftermath, mass displacements in and outside the country. This highlights the salient fact that majority of Afghans are into farming, and at least 58.08% of the land in the country is used for agricultural purposes [18].

Another major contributor to food insecurity is internal displacement. These displacements happen because of storms, floods, droughts, and most importantly, conflicts and violence. In 2020, it has been observed that 3.5 million people were internally displaced, with over 404,000 of them associated with the surge in violence [19]. This number shows an 18% increase compared to the previous year. It is a mistake to brush off the impact of the pandemic as the fatal virus reversed the modest improvements in access to food—efforts that mostly targeted relieving the tragedy after the severe droughts of 2018 [15].

## Recommendations

In the short run, the government should increase the ratio of budget directed towards food security and other areas. With this already generous amount of assistance, the question of what could have gone wrong is omnipresent. The fact is that COVID-19 paralyzed the allocation of aid and food security. Thus, the major priority must be to restrain the spread of the virus, a recommendation highlighted as of imminent importance by the United Nations Development Program (UNDP) [20].

With the rise of the conflict and a huge area of agricultural land being affected by violence, in the short run, the Ministry of Agriculture, Irrigation, and Livestock (MAIL) should implement strategies to maximize the usage of land in the country. While most of the farming land is not accessible, hydroponic farming should be seen as an alternative to invest on. At times when drought is looming, the government should put a ban on the usage of potable water for farming and should ponder drip irrigation to make sure the agricultural sector is not crippled when required.

Another priority Afghanistan, particularly the government, should address is weaning off from being heavily reliant on foreign aid and assistance. This assistance to Afghanistan from different countries may continue, but should be managed well and should focus on the idea that the long-term solution is hinged on making the country reach a level of self-sufficiency in food security. The Food and Agriculture Organization of the United Nations is on the same side of the argument in addition to adding importance on efficient means of agricultural and food products transportation [21]. Land should also be well-managed as most of the land of the country is already used for agriculture so as to shrink the importation levels year by year.

## Conclusion

Afghanistan, a country already suffering from decades of problems, does not have a robust framework of solutions on food security being implemented especially with its low budget and the wide range of challenges. The food crisis in Afghanistan is one of the most crucial issues that requires immediate solutions. Multiple factors adversely influenced food security thus hindering the progress made in improving the situation; and even worsening it. The most recent factors include COVID-19, drought and climate change, conflict and the drastic decline in international aid as a result of the collapse of the government and the Taliban takeover of the country. The country is in urgent need for humanitarian aid from the international community to overcome food insecurity and eventual hunger and poverty of the population.

## Data Availability

Not applicable.

## Abbreviations

COVID-19: Novel coronavirus disease
GDP: Gross domestic product
UN: United Nations
FSAC: Food Security and Agriculture Cluster
IDA: International Development Association
UNDP: United Nations Development Program
MAIL: Ministry of Agriculture, Irrigation, and Livestock
